# Electroclinical characteristics and therapies of tonic spasms

**DOI:** 10.1186/s42494-024-00158-3

**Published:** 2024-08-01

**Authors:** Xi Peng, Yangmei Chen, Zezhi Wang, Xinbo Zhang, Bi Wang, Lang Jin, Xiaoli Wang, Na Yuan, Xiaojing Hu, Xiaomu Wang, Yonghong Liu

**Affiliations:** 1 Department of Neurology, Xijing Hospital of the Air Force Military Medical University, Xi’an, Shaanxi 710032 China; 2 Department of Neurology, The Second Affiliated Hospital of Chong Qing Medical University, Chongqing, 400010 China

**Keywords:** Epilepsy, Tonic spasm, Electroencephalography

## Abstract

**Backgroud:**

Epileptic spasms followed by a tonic component have been frequently observed in patients with late-onset spasms (LOS). However, there is a lack of comprehensive analysis and summary of clinical data related to tonic spasms (TS), including seizures, video-electroencephalogram (V-EEG), synchronous electromyography (EMG) and follow-up data.

**Methods:**

To investigate the characteristics of TS, we prospectively collected the clinical data, including 24-h V-EEG and synchronous EMG data of 32 enrolled patients who suffered from epileptic spasms followed by a TS onset at least once during the 24-h V-EEG in the epilepsy center of Xijing Hospital between June 2015 and July 2020. The patients were prescribed anti-seizure medications (ASMs) and followed up for 2–7 years.

**Results:**

The average age of epilepsy onset was 48.06 ± 16.07 months (range: 25 to 88 months). Among the enrolled patients, 22 patients presented with mild intellectual deficits. During the 24-h video-EEG monitoring, an average of 6.94 TS events (range: 3 to 21) were recorded, and these TS seizures often occurred in clusters. In addition to TS, 26 patients experienced generalized tonic-clonic seizures (GTCS), atypical absence seizures, myoclonic seizure, and epileptic spasms. None of the 32 patients with TS displayed hypsarrhythmia during the 24-h video-EEG recording. A total of 28 patients showed normal EEG backgrounds. Interictal epileptic discharges, including slow waves (SW), spike/sharp slow waves (SSW), and spikes, often displayed multifocally. Notably, two patients achieved seizure freedom for more than 2 years through monotherapy with oxcarbazepine (OXC), which was associated with normalization of the EEG.

**Conclusions:**

It is difficult to classify the patients with TS as any existing epileptic syndromes, which were distinct from West syndrome or Lennox-Gastaut syndrome. TS might be an underreported seizure type and further studies are needed to gain a more comprehensive understanding of the electro-clinical features and appropriate choice of ASMs for treating tonic spasms.

**Supplementary Information:**

The online version contains supplementary material available at 10.1186/s42494-024-00158-3.

## Background

Epileptic spasms are a main type of seizure associated with West syndrome (WS), characterized by short contractions involving axial and proximal muscles [1]. Tonic seizures are another seizure type frequently seen in Lennox-Gastaut syndrome (LGS), a disorder characterized by mixed seizure types, developmental delay, and slow spike-wave complexes during EEG recordings [2, 3].

Recently, epileptic spasms occurring after the first year of life, named late-onset spasms (LOS), have received much attention. Many studies have proposed that LOS has distinct clinical and electrographic characteristics from WS and LGS, and may be a new type of epilepsy syndrome [4–9]. Among the reported cases of LOS, we noted a series of patients showing a tonic component in spasms. De Menezes and Rho [5] reviewed 26 cases of LOS and found that 8 cases had a tonic phase after spasm onset. Eisermann et al. [6] reported 22 cases of cryptogenic LOS, among whom 12 cases showed spasms with a tonic component. Nordli et al. reported 5 of 10 LOS cases with a tonic component [7]. Auvin et al. [4] reported 6 of 19 LOS cases with a tonic component. Ishikawa et al. [8] analyzed 8 symptomatic LOS cases and emphasized that 4 of them had spasms with a tonic phase. As early as 1994, tonic spasm seizures were first reported and named by Fosco and Vigevano [9] as “tonic spasms (TS)”. In 2015, the term “tonic spasm” in LOS was proposed again by Marchi et al. in a case report of a 5-year-old boy [10]. It seems that TS cannot be easily classified as an epileptic spasm, tonic seizure, or any existing seizure type. The clinical and electroencephalogram (EEG) characteristics of TS have rarely been analyzed and summarized separately. In this study, we collected data of patients with TS in the epilepsy center to analyze their clinical, EEG and synchronous electromyography (EMG) patterns, as well as their treatments.

## Methods

### Participants

This was a single-center, prospective study. Information of TS in patients was obtained from June 2015 to July 2020 at the epilepsy center of Xijing Hospital-Air Force Military Medical University, Xi'an, China.

The patient inclusion criteria were: (1) one or more epileptic spasms followed by a tonic seizure as monitored during 24-h video-EEG (V-EEG); (2) ictal EEG and EMG were conducted in every TS seizure according to the definition and classification of epilepsy and epileptic seizure by the International League Against Epilepsy Commission. The following criteria were used to confirm the diagnosis of spasms: (1) each event was characterized by a sudden contraction of proximal limb and axial musculature lasting 1–3 s; (2) EEG patterns included slow-wave transient and/or attenuation of the background; and (3) time-locked EMG recording of deltoid or cervical muscles showed muscle activity characterized by a brief 1–2-s crescendo–decrescendo pattern, often but not always with a diamond-shaped configuration. Tonic seizures are characterized by a gradual increase in EMG activity during muscular contraction, peaking at 3–7 s, and with a sustained EEG discharge frequency of 10–20 Hz. The exclusion criteria of participants were: (1) patients' parents or legal caregivers refused to participate in the study, (2) inability of patients to complete the study, and (3) loss of follow-up. Three technicians and two physicians reviewed and analyzed the EEG data and determined the ictal EEG and EMG of spasm and the following tonic seizure in every TS episode.

### Collection and follow‑up of clinical data

Baseline information was collected by face-to-face interviews of all patients and at least one of their relatives. The baseline information included demographic data, age at seizure onset, seizure type at onset, family history of epilepsy, history of febrile convulsions, physical status, intellectual level and treatment. Follow-up was scheduled as monthly for telephone and every six months for face-to-face interviews. Additional follow-ups allowed according to the needs of the patients or their families, were carried out by face-to-face interviews or telephone calls. Informed consent was signed by patients or their legal guardians.

All patients were evaluated by regular brain magnetic resonance imaging (MRI), and 24-h V-EEG monitoring. The gene examinations and metabolic analyses were conducted in accordance with the guidance provided by the attending epileptologist, taking into account the clinlical data of each individual patient.

The 24-h V-EEG monitoring was performed in an illuminated room with a 32-channel digital video-EEG system (NicoletOne; America and Nihon Kohden; Japan; Bio-logic; America) with scalp electrodes placed according to the international standard lead 10–20 system. The reference electrode was placed at Cz′, a location 1 cm behind Cz. Diffusion tensor imaging (DTI) was performed on a 3 T MRI scanner (Magnetom Trio A Tim System; Siemens, Erlangen, Germany) with a 32-channel phased array head coil. The fractional anisotropy (FA) values of the bilateral subcortical white matter in the frontal, temporal, occipital and parietal areas, as well as the internal capsule and external capsule were analyzed.

The cognitive performace of each patient was assessed with the Wechsler Intelligence Scales for Children and Wechsler Preschool and Primary Scale of Intelligence.

Statistical analysis was performed by using the SPSS, Version 11.5 software (IBM, Armonk, New York). Significance was set at *P* < 0.05.

## Results

Of the 25,256 possible epilepsy patients who received 24-h V-EEG monitoring, 32 cases experienced episodes of TS for one or more times. The clinical features of the 32 patients are summarized in Table 1. The average age of epilepsy onset was 48.06 ± 16.07 months (range: 25 to 88 months). The 32 patients comprised of 18 cases with symptomatic TS (encephalitis in 11 patients and focal cortical dysplasia in 7 patients), and 14 cases with cryptogenic TS. The 18 cases of symptomatic TS had an average epilepsy onset age of 40.33 ± 11.52 months (25 to 58 months), and their physical examinations showed negative results. Ten of them had intellectual deficits. The 14 cases with cryptogenic TS had an average epilepsy onset age of 58.00 ± 15.92 months (26 to 88 months). All their physical examinations, brain MRIs and metabolic analyses showed negative results. The psychomotor development before epilepsy was normal in all patients. With disease progression, 22 patients presented with mild intellectual deficits. Twenty-six patients also experienced other types of seizures besides TS, such as generalized tonic clonic seizures (GTCS), atypical absence seizures, myoclonic seizures, and epileptic spasms.

**Table 1:** Clinical features of 32 patients with tonic spasm

Case	Sex	Age at epilepsy onset (months)	Etiology	Family history	History of febrile convulsion	Epilepsy gene examination	Psychomotor development before epilepsy onset	Psychomotor development during epilepsy	Seizure types	The number of TS recorded by EEG	ASMs	Epilepsy control
1	M	38	Symptomatic	No	No	Negative	Normal	MID	GTCS; TS; AS	5	VPA; OXC	Partially controlled
2	M	26	Symptomatic	No	No	NA	Normal	Normal	TS	3	OXC	Seizure free
3	M	51	Symptomatic	No	No	NA	Normal	MID	GTCS; TS; MS	10	VPA; OXC; LEV	Refractory epilepsy
4	F	53	Cryptogenic	No	No	Negative	Normal	MID	GTCS; TS	7	OXC; TPM	Partially controlled
5	M	88	Cryptogenic	No	No	NA	Normal	Normal	TS	4	OXC	Seizure free
6	M	52	Cryptogenic	No	No	NA	Normal	MID	GTCS; ES; TS	11	OXC; TPM	Partially controlled
7	F	45	Symptomatic	No	No	NA	Normal	MID	TS	9	VPA; OXC; LEV	Refractory epilepsy
8	M	40	Cryptogenic	No	No	NA	Normal	MID	GTCS; ES; TS	6	VPA	Partially controlled
9	M	53	Symptomatic	No	No	NA	Normal	MID	GTCS; MS; TS	8	OXC; TPM	Partially controlled
10	F	25	Symptomatic	No	No	Negative	Normal	MID	TS	20	OXC; TPM LEV	Refractory epilepsy
11	M	35	Symptomatic	No	No	NA	Normal	MID	GTCS; TS	6	VPA; OXC	Partially controlled
12	M	46	Cryptogenic	No	No	NA	Normal	Normal	GTCS; TS	5	VPA; OXC	Partially controlled
13	F	58	Cryptogenic	No	No	NA	Normal	MID	GTCS; TS	7	TPM; OXC	Partially controlled
14	M	68	Cryptogenic	No	No	NA	Normal	MID	GTCS; ES; TS	4	TPM; LEV	Partially controlled
15	M	37	Symptomatic	No	No	Negative	Normal	MID	GTCS; TS; AS	21	TPM; VPA; LTG	Refractory epilepsy
16	M	67	Cryptogenic	No	No	NA	Normal	MID	GTCS; TS;	4	TPM;VPA	Partially controlled
17	M	25	Symptomatic	No	No	Negative	Normal	Normal	TS; AS	3	TPM; VPA	Partially controlled
18	M	56	Symptomatic	No	No	NA	Normal	MID	GTCS; TS; MS	6	VPA; OXC	Partially controlled
19	M	48	Symptomatic	No	No	NA	Normal	Normal	GTCS; TS;	6	LTG; TPM	Partially controlled
20	F	29	Symptomatic	No	No	NA	Normal	Normal	GTCS; TS; AS	9	LTG; TPM	Partially controlled
21	M	67	Cryptogenic	No	No	NA	Normal	MID	GTCS; TS; MS	6	VPA	Partially controlled
22	F	70	Cryptogenic	No	No	NA	Normal	MID	GTCS; AS; TS	7	LTG; TPM	Partially controlled
23	M	68	Cryptogenic	No	No	Negative	Normal	MID	GTCS; TS	4	TPM;LEV	Partially controlled
24	M	45	Symptomatic	No	No	Negative	Normal	MID	GTCS; TS; AS	5	VPA; LEV	Partially controlled
25	M	57	Symptomatic	No	No	NA	Normal	Normal	GTCS; TS	3	VPA; TPM	Partially controlled
26	M	36	Symptomatic	No	No	NA	Normal	Normal	TS	4	VPA; OXC	Partially controlled
27	M	30	Symptomatic	No	No	NA	Normal	Normal	GTCS; TS; AS	6	VPA; OXC	Partially controlled
28	F	67	Cryptogenic	No	No	Negative	Normal	MID	GTCS; TS; MS	7	TPM; LEV	Partially controlled
29	M	58	Symptomatic	No	No	NA	Normal	MID	GTCS; TS;	5	VPA; OXC	Partially controlled
30	M	42	Cryptogenic	No	No	NA	Normal	MID	GTCS; ES; TS	8	OXC; TPM	Partially controlled
31	M	26	Cryptogenic	No	No	Negative	Normal	MID	GTCS; AS; TS	4	OXC; VPA	Partially controlled
32	F	32	Symptomatic	No	No	Negative	Normal	Normal	TS	9	LEV; OXC	Partially controlled

*Abbreviations: ASMs* Anti-seizure medications, *AS* Absence seizure, *D* Day, *ES* Epileptic spasm, *F* Female, *GTCS* Generalized tonic-clonic seizure, *M* Male, *MID* Mild intellectual deficits, *MS* Myoclonic seizure, *NA* Not applicable, *TS* Tonic spasm, *VPA* Valproic acid, *OXC* Oxcarbazepine, *TPM* Topamax, *LEV* Levetiracetam

### Seizures and ictal EEG characteristics

**Fig. 1:**
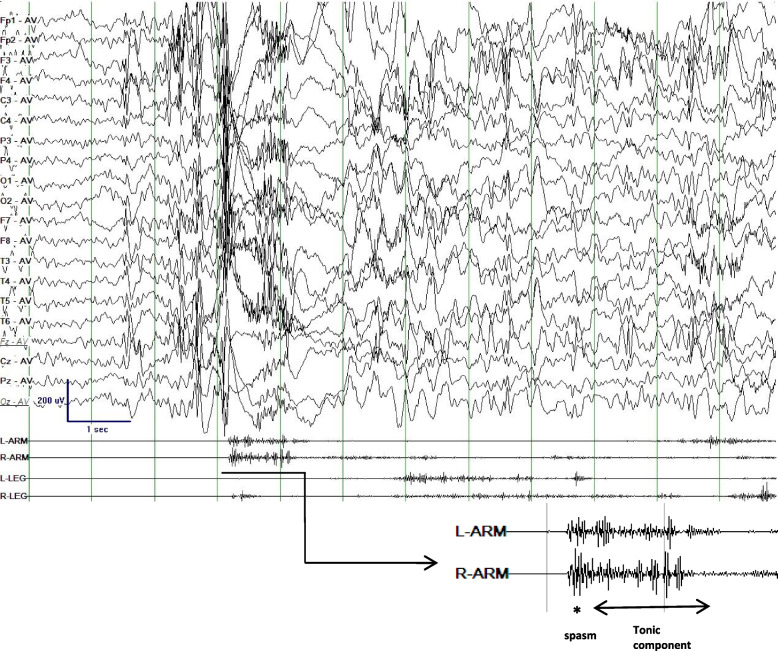
Ictal EEG and EMG of case 1

**Fig. 2:**
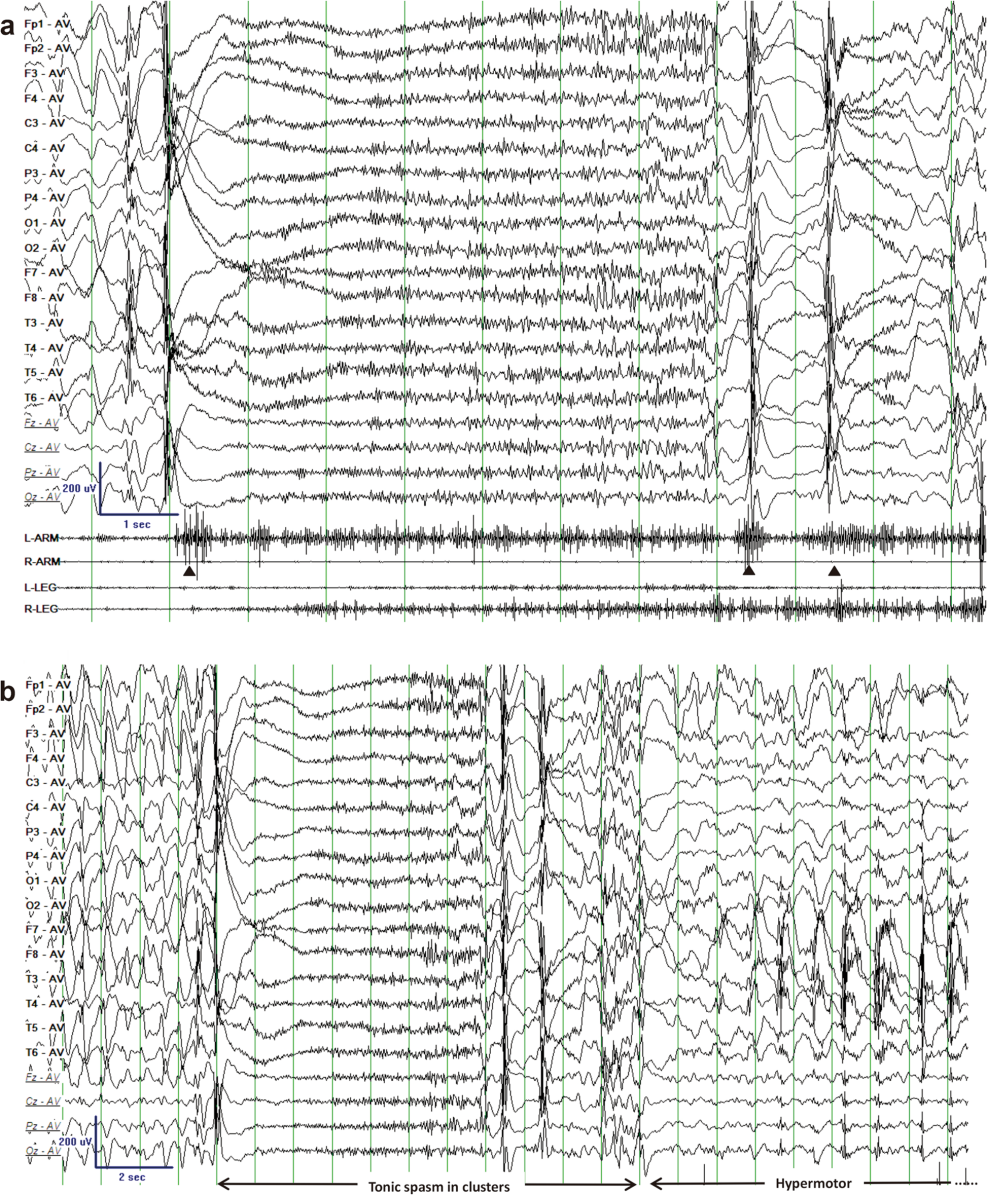
Ictal EEG of case 5. **a**: EEG and EMG patterns of tonic spasm in clusters, a triangle indicates a spasm onset, timebase, 30 mm/s. **b**: EEG pattern of hypermotor following tonic spasm, timebase, 15mm/s

The 222 TS seizures of 32 patients were monitored during 24-h V-EEG. Similar to spasm, TS often occurred in clusters. The average number of tonic spasms monitored during 24-h V-EEG was 6.94 (range, 3–21). The ictal EEG and synchronous EMG are summarized in Table 2. All cases showed the seizure type characterized by an epileptic spasm followed by a tonic component with different durations (Figs. 1 and 2). The initial symptoms of TS were similar across the patients, including sudden rise of arms, shrugging shoulders, flexing neck, with/without eye staring upward. Synchronous EMG showed a rhombic or diamond shape on deltoid EMG as a typical EMG change of spasm. The average duration of EMG was 1843.38 ± 13.57 ms. Synchronous EEG showed a sudden high-voltage slow wave (HVS) (Fig. 2), or deflecting voltage after a sudden high-voltage (HV) spike slow wave or a HV spike wave (Fig. 1). Then, the spasm developed into the tonic phase, in which the EMG showed constant contraction of the deltoid muscle, with average EMG duration of 3512.23 ± 15.53 ms. EEG revealed fast activity with or without electrodecrement. The finishing of the EMG tonic component was accompanied by hands down, and the synchronous EEG showed a postictal pattern (Table 2). Synchronous EMG showed asymmetric muscle contraction at spasm and tonic phases in all patients except one (case 3). Thirty patients had a clustered TS mode (Fig. 2). Eight patients (cases 4, 5, 6, 13, 14, 15, 29, 32) experienced hypermotor movement lasting 10–30 s following TS (Fig. 2b). The hypermotor movement in case 4 and case 32 was displayed as kneeling on the bed and leaning forward with roaring; case 5 and case 29 showed constant kicking on the bed, straight arms with hands groping and smacking lips; and the hypermotor movement in cases 6, 13, 14 and 15 was displayed as standing on the bed, jumping, and tearing things. Videos and quadricep EMGs showed that 29 patients had various intensities of muscle contraction of the legs during TS, while the other 3 patients (cases 1, 3, and 22) did not show obvious muscle contraction of the legs. Both the clinical manifestation and the EEG of TS were stereotypical in our cases. Our patients also had other seizure types that coexisted with TS. TS occurred several months or years after other seizure types in 26 patients, and the secondarily generalized tonic-clonic seizure was usually the first symptom described by the parents of the patients. In the other 6 patients, TS was the only seizure type with no presence of other seizure types.

**Table 2:** EEG and synchronous EMG characteristics of 32 patients with tonic spasm

Case	Age at video EEG (months)	Clusters of stereotyped	TS onset occasion	1 s EEG before TS onset	TS Ictal EEG (spasm)	TS Ictal EEG (tonic phase)	Hyper motor after TS; seconds	Postictal EEG; seconds	Deltoids EMG in TS: L/R	Quadriceps EMG in TS: L/R	EEG Background	Interictal epileptiform discharges
1	121	NA	NREM	SSW	DV after HV-SSW	FA without electrodecrement; 1–2S	NA	HVS/S; 38–50S	+ / + +	-/-	9–10 Hz	Multifocal SSW/SW; dominant in B–F
2	39	NA	Waking and NREM	No special change	HV-SSW	FA without electrodecrement; 2–3S	NA	HVS/S; 50–60S	-/ + +	+ / +	7.5–8 Hz	Multifocal SSW/SW /S; dominant in L–F, T
3	172	NA	NREM	SSW	DV after HV-SSW	FA without electrodecrement; 2–3S	NA	HVS/SSW 3–4S	+ + / + +	-/-	8–9 Hz	Multifocal SSW/SW /S; dominant in B–F
4	134	NA	Waking	No special change	DV after HVS	FA with electrodecrement; 7–8S	SW/SSW; 16–20S	Background	+ / + +	-/ + +	7.5–9 Hz	Multifocal SSW/MSSW; dominant in B–F
5	113	2–3 times/seizure	Waking	HVS superimposed by low-voltage FA	DV after HV-SSW	FA with electrodecrement; 6–7S	SW/SSW; 20–30S	SW/SSW; 13–20S	+ + / +	+ / +	8–10 Hz	Multifocal SSW; dominant in B–F
6	72	NA	Waking and NREM	HVS	DV after HV-SSW	FA with electrodecrement; 6–7S	SW/SSW; 10–20S	SW/SSW; 20–30S	+ + / +	+ / +	6–7 Hz (slower than normal range)	Multifocal SSW/SW; dominant in B–F
7	119	NA	NREM	SSW	DV after HV-SSW	FA without electrodecrement; 2–3S	NA	HVS/S; 40–50S	+ + / + +	-/ +	8–9 Hz	Multifocal SSW/SW; dominant in B–F
8	41	NA	NREM	No special change	DV after HV-SSW	FA without electrodecrement; 3–4S	NA	HVS/S; 50–60S	+ / + +	+ / +	7–9 Hz	Multifocal SSW/SW /S; dominant in R–F, T
9	169	NA	Waking and NREM	No special change	DV after HV-SSW	FA without electrodecrement; 1–2S	NA	HVS/SSW 4–5S	+ + / + +	+/-	7.5–10 Hz	Multifocal SSW/SW /S; dominant in L–F, T
10	137	NA	Waking	HVS	HV-SSW	FA without electrodecrement; 1–2S	NA	SW/SSW; 15–20S	+ / + +	-/ +	8–9 Hz	Multifocal SSW/MSSW; dominant in B–F
11	105	NA	Waking	SSW	DV after HV-SSW	FA without electrodecrement; 3–4S	NA	SW/SSW; 18–30S	+ + / + +	+ + / +	8–11 Hz	Multifocal SSW; dominant in B–F
12	80	NA	Waking	HVS	DV after HV-SSW	FA without electrodecrement; 2–3S	NA	Background	+ + / +	+/-	6–8 Hz (slower than normal range)	Multifocal SSW/SW; dominant in L–F, T
13	110	NA	NREM	HVS	DV after HV-SSW	FA with electrodecrement; 7–8S	SW/SSW; 15–20S	SW/SSW; 10–20S	+ + / +	-/ +	9–11 Hz	Multifocal SSW/SW; dominant in B–F
14	51	2–4 times/seizure	NREM	SSW	DV after HV-SSW	FA with electrodecrement; 6–7S	SW/SSW; 20–30S	SW/SSW; 18–30S	+ / + +	+ / +	7–8 Hz	Multifocal SSW/SW /S; dominant in B–F
15	150	3–4times/seizure	Waking and NREM	HVS	DV after HV-SSW	FA without electrodecrement; 2–3S	SW/SSW; 12–20S	HVS/S; 45–50S	+ + / + +	+/-	8–9.5 Hz	Multifocal SSW/SW /S; dominant in B–F
16	157	NA	NREM	SSW	DV after HV-SSW	FA without electrodecrement; 3–4S	NA	Background	+ / +	-/ +	8–9 Hz	Multifocal SSW/MSSW; dominant in L–F, T
17	90	NA	Waking	No special change	DV after HV-SSW	FA without electrodecrement; 1–2S	NA	HVS/S; 38–50S	+ + / + +	+ / +	8–10 Hz	Multifocal SSW; dominant in B–F
18	95	NA	NREM	No special change	DV after HV-SSW	FA without electrodecrement; 1–2S	NA	HVS/S; 50–60S	+ / + +	+ / +	8–9.5 Hz	Multifocal SSW/SW; dominant in B–F
19	101	NA	NREM	No special change	DV after HV-SSW	FA with electrodecrement; 7–8S	NA	HVS/SSW 3–4S	+ / + +	+ / +	9–10 Hz	Multifocal SSW/SW /S; dominant in L–F, T
20	60	NA	Waking	SSW	HV-SSW	FA with electrodecrement; 6–7S	NA	HVS/SSW 5–6S	+ / + +	+ / +	9–10 Hz	Multifocal SSW/SW /S; dominant in B–F
21	161	NA	NREM	SSW	HV-SSW	FA with electrodecrement; 6–7S	NA	Background	+ + / + + +	-/ +	8.5–9 Hz	Multifocal SSW/MSSW; dominant in B–F
22	145	NA	NREM	HVS	DV after HV-SSW	FA with electrodecrement; 7–8S	NA	Background	+ / + +	-/-	9–10 Hz	Multifocal SSW; dominant in B–F
23	108	2–5 times/seizure	NREM	No special change	DV after HV-SSW	FA with electrodecrement; 6–7S	NA	HVS/S; 35–50S	+ + / + +	+ + / +	8–9 Hz	Multifocal SSW/SW; dominant in B–F
24	73	NA	Waking and NREM	HVS	DV after HV-SSW	FA without electrodecrement; 1–2S	NA	HVS/S; 22–65S	+ + + / + +	+ / +	9–10 Hz	Multifocal SSW/SW; dominant in B–F
25	125	NA	NREM	No special change	DV after HV-SSW	FA without electrodecrement; 1–2S	NA	HVS/SSW 2–6S	+ + / + +	-/ +	6–8 Hz (slower than normal range)	Multifocal SSW/SW /S; dominant in R–F, T
26	35	NA	Waking	SSW	DV after HV-SSW	FA without electrodecrement; 2–3S	NA	SW/SSW; 15–25S	+ / + +	+/-	9–11 Hz	Multifocal SSW/SW /S; dominant in B–F
27	172	NA	Waking	No special change	DV after HV-SSW	FA without electrodecrement; 1–2S	NA	SW/SSW; 15–30S	+ / + +	+/-	9–10 Hz	Multifocal SSW/MSSW; dominant in B–F
28	135	NA	Waking	HVS superimposed by low-voltage FA	DV after HV-SSW	FA without electrodecrement; 1–2S	NA	Background	+ / + +	-/ +	7.5–9 Hz	Multifocal SSW; dominant in B–F
29	112	NA	Waking and NREM	HVS	DV after HV-SSW	FA without electrodecrement; 2–3S	SW/SSW; 15–20S	SW/SSW; 10–25S	+ + / + +	+ + / +	8.5–9 Hz	Multifocal SSW/SW; dominant in B–F
30	73	NA	Waking	No special change	DV after HV-SSW	FA with electrodecrement; 6–7S	NA	SW/SSW; 18–30S	+ + / +	-/ +	7–9 Hz	Multifocal SSW/SW; dominant in B–F
31	101	NA	Waking	SSW	HV-SSW	FA with electrodecrement; 7–8S	NA	SW/SSW; 10–25S	+ / + +	+/-	8–11 Hz	Multifocal SSW/SW /S; dominant in R–F, T
32	60	2–3 times/seizure	Waking	No special change	DV after HV-SSW	FA with electrodecrement; 6–7S	SW/SSW; 10–20S	SW/SSW; 20–30S	+ / + +	+ / +	6–7 Hz (slower than normal range)	Multifocal SSW/SW /S; dominant in B–F

*Abbreviations: B* Bilateral, *DV* Deflecting voltage, *F* Frontal area, *FA* Fast activity, *HV* High-voltage, *HVS* High-voltage slow wave, *L* Left, *R* Right, *MSSW* Multiple spike and slow wave complex, *NA* Not applicable, *NREM* Non-rapid eye movement sleeping, *S* Spike, *SSW* Spike/sharp slow wave, *SW* Slow wave, *T* Temporal area, *TS* Tonic seizure

### Interictal EEG data

**Fig. 3:**
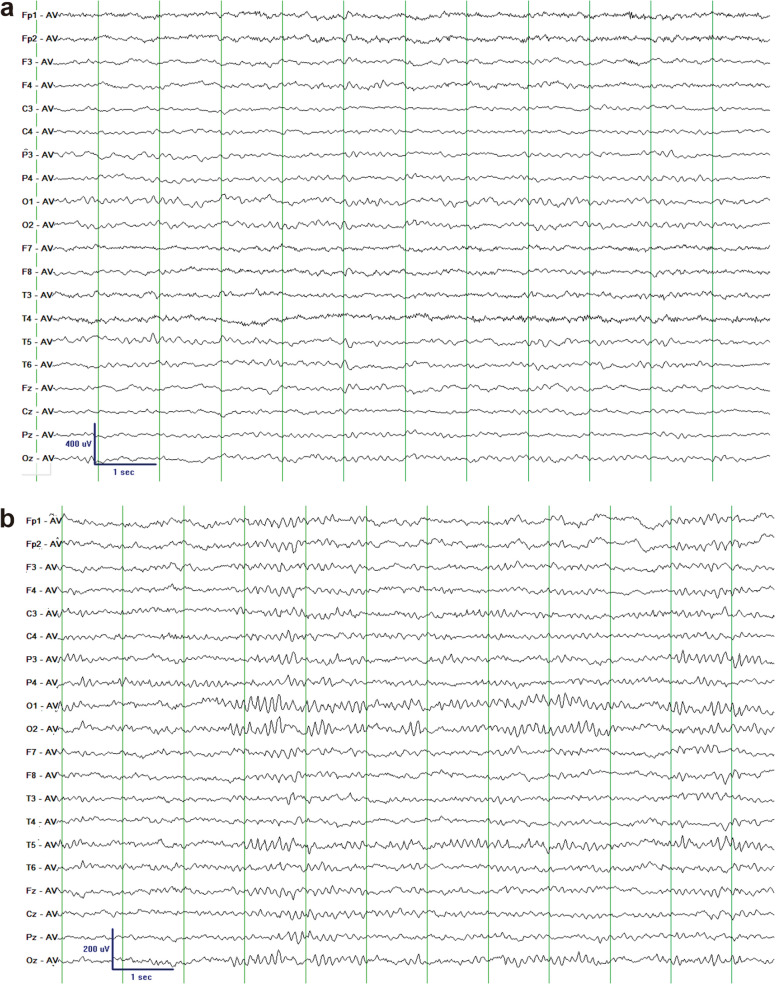
Background EEG of case 2 (**a**) and case 3 (**b**) in awaking stage showed normal activities

**Fig. 4:**
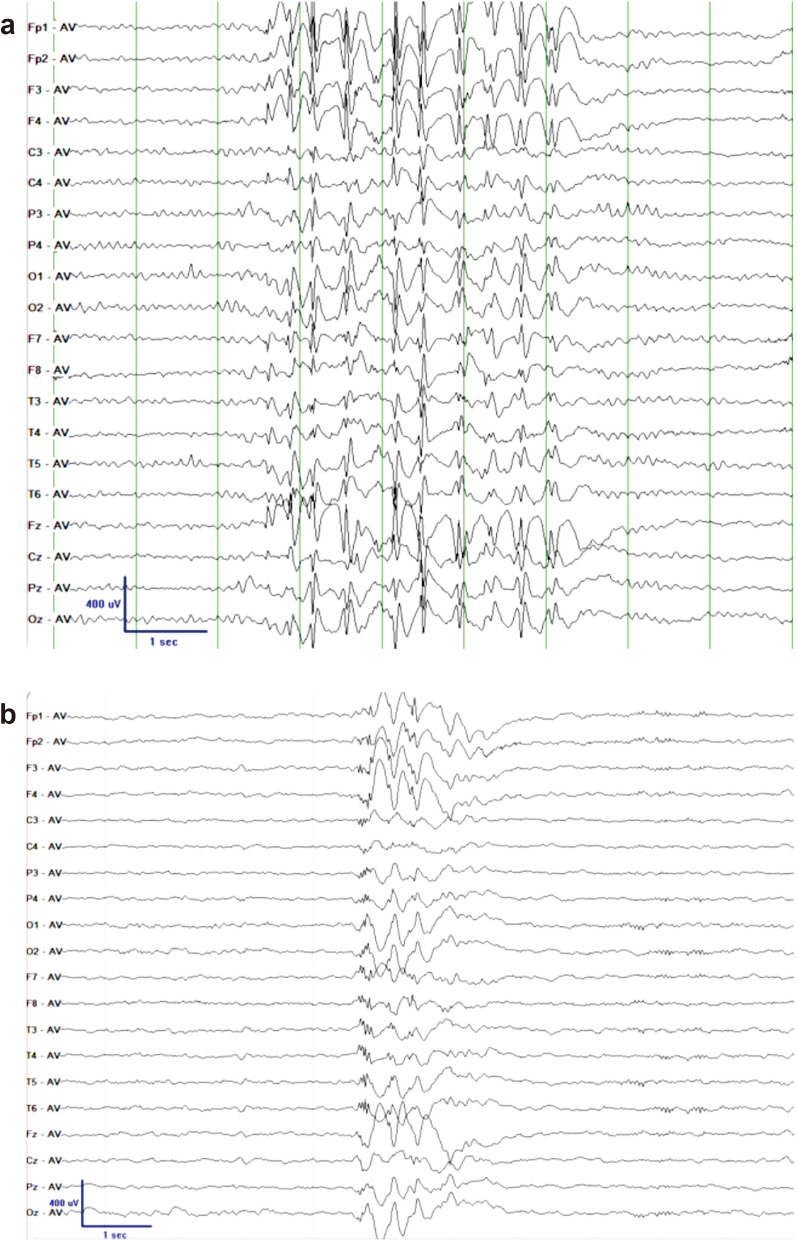
Epileptic discharges of interictal EEG in case 3 (**a**: awaking stage) and case 4 (**b**: NREM sleeping stage)

Hypsarrhythmia was not found in the interictal EEGs of the 32 TS patients. A total of 28 patients showed normal EEG backgrounds (Fig. 3); cases 6, 12, 25 and 32 had a slower background. Interictal epileptic discharges, including slow waves (SW), spike/sharp slow wave (SSW) and spikes, were always found to be multifocal with a frontal dominance in 24 patients (Fig. 4). Eight patients (cases 2, 8, 9, 12, 16, 19, 25, and 31) showed dominance in both frontal and temporal areas (Table 2).

### DTI

Only one patient (case 4) agreed to receive DTI examination. There was no significant difference in the FA value between the left and the right hemispheres in each examined area.

### Course of the disease

The time of data collection spanned five years, containing at least two years of follow up. Two patients were seizure-free for more than two years together with normalization of EEG with monotherapy of oxcarbazepine (OXC). Twenty-six patients were partially controlled for more than one year with obvious decreased frequency and degree of seizures with the use of the following anti-seizure medications (ASMs): OXC, or a combination of valproic acid (VPA), lamotrigine (LTG), levetiracetam (LEV) and topiramate (TPM). Four patients turned into refractory epilepsy after combined treatment with three ASMs (Table 1).

## Discussion

LOS has received much attention these years. Whether LOS should be considered as a new epileptic syndrome or not has been discussed in many studies [4–8]. However, in this study we focused on a special type of seizure that has been frequently found in LOS cases: a spasm followed by a tonic component. This type of seizure seems to have distinct clinical and EEG characteristics from other seizure types; thus, was named as “tonic spasm”. All patients in our study had an onset age over 2 years. The morbidity rate in our study was approximately 0.2% in children and infants diagnosed with epilepsy.

Tonic spasms in our cases were often characterized by starting with a sudden flexion epileptic spasm, manifesting as a specific posture, such as raising arms, buckling neck, and shrugging shoulders, and the synchronous EMG showed a rhombic or diamond shape on the deltoid EMG. The duration of spasms in EMG lasted 1843.38 ± 13.57 ms, mostly > 1800 ms, which was consistent with, or longer than, the duration of spasm seizures occurring alone. Videos and quadricep EMGs revealed that leg movements were not as obvious and typical as arm movements. The spasms were always followed by a tonic component, presented as the posture described above maintained for a few seconds. Synchronous EMG showed persistent contraction of proximal muscles until arms down, suggesting termination of the tonic phase. The manifestations from videos were similar to previous reports [10]. The duration of the tonic period in EMG lasted 3512.23 ± 15.53 ms, mostly > 3000 ms, which was consistent with the definition of tonic seizure alone. The tonic spasm seizures mainly involved proximal or axial muscles. The symptoms, ictal EEG and EMG all indicated the presence of a spasm and a tonic phase in a TS seizure.

Ictal EEG of TS in previous studies often revealed a generalized or diffuse HVS followed by low-voltage fast activities [6, 10]. The EEG features of our cases were similar as previous reports, but there were also differences in both the spasm and the tonic phases: the spasm onset was associated with a deflecting voltage after a sudden HV spike slow wave or HV spike wave in 27 patients; the tonic phase was associated with fast activities without voltage reduction in 19 patients. Therefore, the ictal EEG of TS in this study indicated the presence of two essential elements: (1) a sudden high-voltage wave with or without a following deflecting voltage, which was associated with a spasm; and (2) a phase of fast activities with or without voltage reduction, which was related to the tonic component.

We considered whether the tonic spasm was a seizure form of epileptic encephalopathy, such as WS and LGS, but evidence in our cases indicated it may not be. First, the electroclinical features are distinct from WS and LGS. Second, the background interictal EEG in our cases did not show hypsarrhythmia. Moreover, the patients did not have obvious psychomotor regression. In addition, 28 cases had good therapeutic responses to ASMs, and 2 of whom were completely controlled for more than two years. It is necessary to identify the probability of seizure recurrence with longer follow-up time. The other 26 patients showed good responses to one or two ASMs at the early treatment stage, although they did not become completely seizure-free, which may be due to the inadequate drug doses and patients’ poor compliance. Only four patients developed intractable epilepsy. The normal background EEG of the 22 patients and their good responses to ASMs are in accordance with a previous report that treatment efficiency is associated with EEG background activity changes [11]. In the studies of symptomatic LOS by Ishikawa et al. [8] and Auvin et al. [4], patients displayed severe developmental delays, drug resistance, and temporal or temporofrontal predominance of spike activity. The LOS with/without the tonic component was regarded as an epileptic encephalopathy and may be an intermediate between WS and LGS. A similar situation was observed in the report by Ronzano et al. that symptomatic LOS had worse prognosis and psychomotor regression compared to cryptogenic LOS [12]. In the report by Nordli et al. [7], one patient with cryptogenic TS had a good response to adrenocorticotropic hormone (ACTH), while two patients with symptomatic TS failed to respond to ASMs. There are some obvious differences between cryptogenic and symptomatic TS in psychomotor development and ASM response. Our cases showed frontal instead of temporal predominance of epileptiform discharges, suggesting that the cryptogenic TS may have different pathologic structure from symptomatic TS and mainly involves the frontal area. In the study of cryptogenic LOS by Eisermann et al. [6], the tonic component is more frequently found in children that responded to treatment, and none of the patients developed LGS within the observation period. Our study had some difference with the above report. The disease course and EEG pattern of our cases suggested that TS may be a unique seizure type in cryptogenic LOS usually with a good response to ASMs, rather than being an intermediate between WS and LGS. We considered that cryptogenic etiology combined with a tonic component in LOS may predict a good prognosis; however, further studies with a larger sample size are needed to confirm this assumption.

The sequential occurrence of different epileptic seizure types in an epileptic episode is common. We show here 8 patients with hypermotor seizures following TS. The spasm, tonic and hypermotor seizure occurred sequentially in one epileptic episode, which has been rarely reported. The different epileptic seizure stereotypes occurring in sequence may indicate that specific brain networks are involved in the specific seizure types. Hypermotor seizures are primarily reported in mesial frontal or orbitofrontal epilepsy [13]. The frontal lobe, including the motor cortex and the supplementary motor area, is responsible for organized, oriented activities. Meanwhile, the interictal EEG of eight hypermotor cases showed multifocal epileptic discharges, predominant in the bilateral frontal regions, suggesting that the frontal lobe is involved in the spasm-tonic-hypermotor seizures. Intracerebral EEG monitoring could be used to confirm the epileptogenic zone for spasm-tonic seizures and spasm-tonic-hypermotor seizures.

DTI was performed in one patient to detect underlying abnormalities in the white matter. Although we did not detect any specific abnormality, we could not deny the roles of focal networks in the pathogenesis. In future case-control studies, more patients and age- and sex-matched healthy controls should be included.

The onset age of tonic spasm seizures was over 1-year-old in previous studies. We observed patients including all infants and children in our center without the above pattern. It is interesting that all TS patients reported here, either cryptogenic or symptomatic, had onset age over two years. Our study suggested that the TS seizures are a unique seizure type that mainly occur in LOS.

Previous studies showed that LOS or epileptic spasms without hypsarrhythmia tend to occur in clusters [5]. TS in clusters were also reported in a 5-year-old boy [1]. Most of the patients in those studies had disorders of the central nervous system. Some patients in our study exhibited the clustering stereotype. A mechanism of clusters is postulated to be the failure of termination of the excitable discharges or increased excitation of neuronal circuits that lead to epilepsy [14]. Recent research revealed that seizure clusters are independently associated with perinatal/congenital brain injury, and intractable epilepsy is at a higher risk of developing seizure clusters [15, 16]. We considered that cryptogenic TS with good ASM responses is a distinct seizure type from epileptic spasms or symptomatic TS, and it may have clusters.

In our study, two patients showed good response to monotherapy of OXC, 14 showed partial control by combination therapy, including OXC. This finding suggested that OXC may be an effective ASM for cryptogenic TS. Other studies suggested that vigabatrin [6, 10] is a good choice for TS, while hydrocortisone [6] and ACTH may be effective for LOS. More samples and pharmacological studies are needed to formulate therapeutic schemes.

In this study, the presence of hypermotor movement [13, 17], the frontal dominance of interictal epileptic discharges, and the responsiveness to OXC in many patients [18] suggest that the TS may be a focal seizure originating from the frontal area. Moreover, tonic limb posturing is recognized as a common form of frontal epilepsy at present [19, 20]. Frontal temporal impairment was described in a 5-year-old case of TS [9] and a 7-year-old case of epileptic spasms without hypsarrhythmia [21]. The majority of interictal epileptic discharges were bilateral, probably because that the frontal seizures often originate from deep frontal structures, such as the medial and base area, which lead to spread of discharges to both sides. Moreover, most of the patients showed asymmetry in TS, indicating that the epileptic discharges may originate from one side and expand quickly to the other side. Further examinations such as positron emission tomography-computed tomography and intracranial EEG may provide some new evidence.

There were some limitations in this study. We started data collection in 2015 when we noticed the first case of this special type of seizure. Therefore, sample size in the study was small, and the follow-up period was not sufficient. Further studies with a longer follow-up period, a larger sample size and more comprehensive examinations would provide more information of this seizure type.

## Conclusions

We identified patients with TS that cannot be classified as any existing seizure type. There were some common clinical and electrophysiology characteristics in the patients. (1) Seizures often started with a sudden flexion epileptic spasm followed by a tonic posture lasting for a few seconds. (2) Ictal EEG displayed a sudden high voltage wave with or without deflecting voltage associated with a spasm, followed by a phase of fast activity related to tonic component. (3) The disease course and the EEG pattern of the patients suggested that TS may be a unique seizure type of LOS. (4) TS occurred with other seizure forms in the same patient. (5) TS occurred commonly in clusters. (6) TS could occur in both awake and NREM sleeping states. (7) TS patients may have good response to OXC, VPA, LTG, LEV or TPM, especially OXC. We assume that TS is a new seizure type. A cryptogenic etiology combined with this seizure type may predict a better prognosis.

## Supplementary Information

**Supplementary Material 1.**

## Data Availability

The data that support the findings of this study are available from the corresponding author upon reasonable request.

## Abbreviations

ACTH: Adrenocorticotropic hormone
ASM: Anti-seizure medication
DTI: Diffusion tensor imaging
EEG: Electroencephalogram
EMG: Electromyography
FA: Fractional anisotropy
GTCS: Generalized tonic clonic seizure
HVS: High-voltage slow wave
HV: High-voltage
LEV: Levetiracetam
LGS: Lennox-Gastaut syndrome
LOS: Late-onset spasm
LTG: Lamotrigine
MRI: Magnetic resonance imaging
OXC: Oxcarbazepine
PET: Positron emission tomography
SW: Slow wave
SSW: Spike/sharp slow wave
TPM: Topiramate
TS: Tonic spasms
WS: West syndrome
