# Supplementary material for: Electroclinical characteristics and therapies of tonic spasms
Source: Acta Epileptol. 2024 Aug 1;6:25. doi: 10.1186/s42494-024-00158-3 (PMC11960261; doi:10.1186/s42494-024-00158-3)
Supplement: Supplementary file 1 — Supplementary Material 1. [file 42494_2024_158_MOESM1_ESM.doc]

**Table S1 Summary of studies reporting patients with tonic spasms**

| **Author/publication year** | **Age group** | **Sample size (*n*)** | **Patients with TS (*n*)** |
| --- | --- | --- | --- |
| DeMenezes et al./2002[1] | 4–17 years | 26 | 8 |
| Eisermann et al./2006[2] | 4–17 years | 22 | 12 |
| Nordli et al./2007[3] | mean age, 9.03 ± 3.69 years | 10 | 5 |
| Auvin et al./2010[4] | 1–12 years | 19 | 6 |
| Ishikawa et al./2014[5] | 13–82 months | 8 | 4 |
| Marchi et al./2015[6] | 5 years | 1 | 1 |
| Our study/2024 | 25–88 months | 32 | 32 |

[1] De Menezes MA, Rho JM. Clinical and electrographic features of epileptic spasms persisting beyond the second year of life. Epilepsia . 2002; 43: 623-30.

[2] Eisermann MM, Ville D, Soufflet C, Plouin P, Chiron C, Dulac O, et al. . Cryptogenic late-onset epileptic spasms: an overlooked syndrome of early childhood? Epilepsia. 2006; 47:1035-42.

[3] Nordli DR Jr, Korff CM, Goldstein J, Koh S, Laux L, Kelley KR. Cryptogenic late-onset epileptic spasms or late infantile epileptogenic encephalopathy? Epilepsia. 2007; 48: 206-08.

[4] Auvin S, Lamblin MD, Pandit F, Vallée L, Bouvet-Mourcia A. Infantile epileptic encephalopathy with late-onset spasms: report of 19 patients. Epilepsia. 2010; 51: 1290-96.

[5] Ishikawa N, Kobayashi Y, Fujii Y, Tajima G, Kobayashi M. Ictal electroencephalography and electromyography features in symptomatic infantile epileptic encephalopathy with late-onset spasms. Neuropediatrics. 2014; 45: 36-41.

[6] Marchi LR, Seraphim EA, Corso JT, Naves PV, Carvalho KC, Ramirez MD, et al. . Epileptic spasms without hypsarrhythmia in infancy and childhood: tonic spasms as a seizure type. Epileptic Disord. 2015; 17: 188-93.
